# On the *p*th moment estimates of solutions to stochastic functional differential equations in the G-framework

**DOI:** 10.1186/s40064-016-2388-x

**Published:** 2016-06-24

**Authors:** Faiz Faizullah

**Affiliations:** Department of Basic Sciences and Humanities, College of Electrical and Mechanical Engineering, National University of Sciences and Technology (NUST), Islamabad, Pakistan

**Keywords:** *p*th Moment estimates, G-Brownian motion, Stochastic functional differential equations, Path-wise asymptotic estimates, Non-linear growth condition

## Abstract

The aim of the current paper is to present the path-wise and moment estimates for solutions to stochastic functional differential equations with non-linear growth condition in the framework of G-expectation and G-Brownian motion. Under the nonlinear growth condition, the *p*th moment estimates for solutions to SFDEs driven by G-Brownian motion are proved. The properties of G-expectations, Hölder’s inequality, Bihari’s inequality, Gronwall’s inequality and Burkholder–Davis–Gundy inequalities are used to develop the above mentioned theory. In addition, the path-wise asymptotic estimates and continuity of *p*th moment for the solutions to SFDEs in the G-framework, with non-linear growth condition are shown.

## Background

Stochastic dynamical systems have a wide range of applications inside as well as outside the field of mathematics. The quantitative studies of different fields such as physics, engineering, ecological sciences, system sciences and medicine have been driven by stochastic dynamical systems. Stochastic differential equations (SDEs) are often used to model financial quantities such as asset prices, interest rates and their derivatives. These equations have become standard models for population dynamics and biological systems. Stochastic functional differential equations (SFDEs) in the G-framework were initiated by Ren et al. (2013). Then studied by Faizullah (2014), he developed the existence-and-uniqueness theorem with Cauchy–Maruyama approximation scheme (Faizullah 2014). Later, he proved the comparison result, with the help of which he established the existence theory for SFDEs in the G-framework with discontinuous drift coefficients (Faizullah et al. 2016). G-expectation, which is a nonlinear expectation, defined by Peng (2006), has been motivated by stochastic volatility problems and risk measures in finance (Gao 2009; Peng 2008, 2010). This led him to derive G-Brownian motion that is a novel stochastic process. Being different from the classical Brownian motion as it is not based on a given particular probability space, G-Brownian motion qualifies itself for a new and extremely rich structure which nontrivially generalizes the classical one. Some of the pertinent stochastic calculus which were established by him included G-Itô’s integral, G-Itô’s formula and G-quadratic variation process $\langle B \rangle$. A new and interesting phenomenon that is related to the G-Brownian motion is the fact that its quadratic variation process, which is also a continuous process, has got stationary and independent increments. Therefore, it continues to qualify for being termed as a Brownian motion. Thus, the idea of G-framework-related stochastic differential equations was initiated (Peng 2006, 2008). Due to the applicability of the theory, many authors published their work on this emerging phenomenon in a short span of time (Bai and Lin 2014; Denis et al. 2010; Xua and Zhang 2009). As important as the existence theory, moment estimate is one of the most useful and basic schemes of analyzing dynamic behavior of SFDEs. It is also worth noting that the *p*th moment of the solution for such SDEs driven by G-Brownian motion with non-linear growth condition has not been fully explored, which remains an interesting research topic. This article will fill the mentioned gap. We present the analysis for the solution to the following SFDE in the G-framework

1 $$\begin{aligned} dY (t) = \kappa (t, Y_{t}) dt+ \lambda (t, Y_{t}) d \langle B, B \rangle (t) + \mu (t, Y_{t}) dB(t),\quad t\in [0,\infty ), \end{aligned}$$

with initial data $Y_{t_0}=\zeta$ satisfying

2 $$\begin{aligned} Y_{t_{0}}&= \zeta = \left\{ \zeta (\theta ){:} - \tau < \theta \le 0 \right\} \; is \; {\mathcal {F}}_{0}{\text{-}}measurable, \; BC ( [ -\tau , 0]; \mathbb {R}^n ){\text{-}} valued \nonumber \\&\quad random \; variable \; such \; that \; \zeta \in M_{G}^{2} \left( [ - \tau , 0] ; \mathbb {R}^n \right) . \end{aligned}$$

It is understood that *Y*(*t*) is the value of stochastic process at time *t* and $Y_{t} = \{ Y(t + \theta ){:}\,-\rho \le \theta \le 0,\rho >0 \},$ indicates $BC([-\rho , 0]; \mathbb {R})$-valued stochastic process, which is a collection of continuous and bounded real valued functions $\varphi$ defined on $[-\rho , 0]$ having norm $\Vert \varphi \Vert =\sup _{ -\rho \le \theta \le 0}\mid \varphi (\theta )\mid$. The coefficients $\kappa ,\,\lambda$ and $\mu$ are Borel measurable real valued functions on $[0, T]\times BC ([-\rho , 0]$ (Faizullah et al. 2016). The rest of the paper is organized as follows: “Preliminaries” section is devoted to some basic definitions and results. “*p*th Moment estimates for SFDEs in the G-framework” section presents the *p*th moment estimates for SFDEs in the G-framework, under non-linear growth condition. “Continuity of *p*th moment for SFDE in the G-framework” section shows that the *p*th moment of solution to SFDE is continuous. The path-wise asymptotic estimates are given in “Path-wise asymptotic estimate” section.

## Preliminaries

In this section some fundamental notions and results are given, which are used in the forthcoming sections of this paper. For more detailed literature of G-expectation, see the papers Denis et al. (2010), Faizullah (2012), Li and Peng (2011), Song (2013) and book Peng (2010).

### **Definition 1**

Let ${\mathcal {H}}$ be a linear space of real valued functions defined on a nonempty basic space $\Omega .$ Then a sub-linear expectation *E* is a real valued functional on ${\mathcal {H}}$ with the following features:

- For all $Y,Z\in {\mathcal {H}}$, if $Y\le Z$ then $E[Y]\le E[Z]$.
- For any real constant $\gamma ,\,E[\gamma ]=\gamma$.
- For any $$\theta >0,\,E[\theta Z]=\theta E[Z]$$.
- For every $$Y,Z\in {\mathcal {H}},\,E[Y+Z]\le E[Y]+E[Z]$$.

Let $C_{b.Lip}(\mathbb {R}^{l\times d})$ denotes the set of bounded Lipschitz functions on $\mathbb {R}^{l\times d}$ and

$$\begin{aligned} L^p_{G}(\Omega _T)=\left\{ \phi (B_{t_1},B_{t_2},\ldots ,B_{t_l}{/}l\ge 1,t_{1},t_{2},\ldots ,t_{l}\in [0,T],\phi \in C_{b.Lip}(\mathbb {R}^{l\times d}))\right\} . \end{aligned}$$

Let $\delta _i\in L_G^p(\Omega _{t_{i}}),\,i=0,1,\ldots ,N-1$ then $M^0_G(0,T)$ denotes the collection of processes of the following type:

$$\begin{aligned} \eta _t(w)=\sum _{i=0}^{N-1}\delta _i(w)I_{[t_i,t_{i+1}]}(t), \end{aligned}$$

where the above process is defined on a partition $\pi _T=\{t_0,t_1,\ldots ,t_N\}$ of [0, *T*]. Associated with norm $\Vert \eta \Vert =\{\int _0^TE[|\eta _u|^p]du\}^{1/p},\,M_{G}^{p}(0,T),\,p\ge 1$, is the completion of $M_{G}^{0}(0,T)$.

### **Definition 2**

Let $(B_t)_{t\ge 0}$ be a *d*-dimensional stochastic process defined on $(\Omega , C_{l,lip}(H),{E}),$ such that $B_0 = 0$. The increment $B_{t+m} - B_t$ is G-normally distributed for any $t,m \ge 0,\,n \in N$ and $0\le t_1\le t_2\le ,\cdots , \le t_n \le t,$ it is independent from $B_{t_1},B_{t_2}, \ldots B_{t_n}.$ Then $(B_t)_{t\ge 0}$ is known as G-Brownian motion.

For every $\eta _t\in M_G^{2,0}(0,T),$ the G-Itô’s integral $I(\eta )$ and G-quadratic variation processes $\{\langle B\rangle _t\}_{t\ge 0}$ are respectively given by

$$\begin{aligned} I(\eta )&=\int _0^T\eta _udB_u=\sum _{i=0}^{N-1}\delta _i(B_{t_{i+1}}-B_{t_i}),\\ \langle B\rangle _t&=B_t^2-2\int _0^tB_udB_u. \end{aligned}$$

We now state three important inequalities known as Hölder’s inequality, Bihari’s inequality and Gronwall’s inequality respectively (Mao 1997).

### **Lemma 3**

*If*$\frac{1}{q}+\frac{1}{r}=1$*for any*$q,r>1,\,g\in L^2$*and*$h\in L^2$*then*$gh\in L^1$*and*

$$\begin{aligned} \int _c^d gh\le \left( \int _c^d |g|^q\right) ^{\frac{1}{q}} \left( \int _c^d |h|^r\right) ^{\frac{1}{r}}. \end{aligned}$$

### **Lemma 4**

*Let*$C\ge 0,\,h(t)\ge 0$*andw*(*t*) *be a real valued continuous function on* [*c*, *d*]. *If for all*$c\le t\le d,\,w(t)\le C+\int _{c}^d h(s)w(s)ds$, *then*

$$\begin{aligned} w(t)\le Ce^{\int _{c}^t h(s)ds}, \end{aligned}$$

*for all*$$c\le t\le d$$.

The following two lemmas are borrowed from the book Mao (1997).

### **Lemma 5**

*Let*$a,b\ge 0$*and*$\epsilon \in (0,1).$*Then*

$$\begin{aligned} (a+b)^2\le \frac{a^2}{\epsilon }+\frac{b^2}{1-\epsilon }. \end{aligned}$$

### **Lemma 6**

*Assume*$p\ge 2$*and*$\hat{\epsilon },a,b>0.$*Then the following two inequalities hold*.

(i) $$a^{p-1}b\le \frac{(p-1)\hat{\epsilon }a^p}{p}+\frac{b^p}{p\hat{\epsilon }^{p-1}}.$$
(ii) $$a^{p-2}b^2\le \frac{(p-2)\hat{\epsilon }a^p}{p}+\frac{2b^p}{p\hat{\epsilon }^{\frac{p-2}{2}}}.$$

### **Theorem 7**

*Let*$Y\in L^p$. *Then for each*$\epsilon >0,$

$$\begin{aligned} \hat{C}(|Y|^p>\epsilon )\le \frac{E[|Y|^p]}{\epsilon }. \end{aligned}$$

In the above Theorem 7, $\hat{C}$ is known as capacity defined by $\hat{C}(H)=\sup _{P\in {\mathcal {P}}}P(H)$, where ${\mathcal {P}}$ is a collection of all probability measures on $(\Omega , {\mathcal {B}}(\Omega )$ and $H\in {\mathcal {B}}(\Omega )$, which is Borel $\sigma$-algebra of $\Omega$. Also, we remind $\hat{C}(H)=0$ means that set *H* is polar and a property holds quasi-surely (*q*.*s*. in short) means that it holds outside a polar set. The rest of the paper is organized as follows. In “Preliminaries” section, *thep*th moment estimates are studied. In “*p*th Moment estimates for SFDEs in the G-framework” section, continuity of *p*th moment is shown. In “Continuity of *p*th moment for SFDE in the G-framework” section, path-wise asymptotic estimates for SFDEs driven by G-Brownian motion are given.

## *p*th Moment estimates for SFDEs in the G-framework

Let Eq. (1) admit a unique solution *Y*(*t*). Assume that a non-linear growth condition holds, which is given as follows. For every $\psi \in BC([-\tau ,0];\mathbb {R}^d)$ and $t\in [0,T]$,

3 $$\begin{aligned} |\kappa (t,\psi )|^2+|\lambda (t,\psi )|^2+|\mu (t,\psi )|^2\le \Upsilon \left( 1+|\psi |^2\right) , \end{aligned}$$

where $\Upsilon (\cdot ){:}\,\mathbb {R}_+\rightarrow \mathbb {R}_+$ is a non-decreasing and concave function such that $\Upsilon (0)=0,\,\Upsilon (x)>0$ for $x>0$ and

4 $$\begin{aligned} \int _{0+}\frac{dx}{\Upsilon (x)}=\infty . \end{aligned}$$

As $\Upsilon$ is concave and $\Upsilon (0)=0$, there exists two positive constants $\alpha$ and $\beta$ such that

5 $$\begin{aligned} \Upsilon (x)\le \alpha +\beta x, \end{aligned}$$

for all $$x\ge 0.$$

### **Theorem 8**

*Assume that the non-linear growth condition* (3) *holds. Let*$E\Vert \zeta \Vert ^p<\infty$*and*$p\ge 2$. *Then*

$$\begin{aligned} E\left[ \sup _{-\tau \le v\le T}|Y(v)|^p\right] \le E\Vert \zeta \Vert ^p+\alpha _3e^{\beta _3T}, \end{aligned}$$

*where*$\alpha _3=T[2\alpha _1(1+c_1) +\alpha _2(c_1(p-1)+pc^2_3)],\,\beta _3=[2\beta _1(1+c_1)+\beta _2(p-1+pc^2_3)],\,\alpha _1=\frac{1}{\hat{\epsilon }^{p-1}}(2)^{\frac{p}{2}-1}[(\alpha +\beta ))^{\frac{p}{2}}+(\beta )^{\frac{p}{2}}E\Vert \zeta \Vert ^p]$*and*$\beta _1=(p-1)\hat{\epsilon }+\frac{(2\beta )^{\frac{p}{2}}}{2 \hat{\epsilon }^{p-1}},\,\alpha _2=\frac{1}{\hat{\epsilon }^{\frac{p-2}{2}}}(2)^{\frac{p}{2}}\left[ (\alpha +\beta )^{\frac{p}{2}}+(\beta )^{\frac{p}{2}}E\Vert \zeta \Vert ^p],\,\beta _2=[(p-1)\hat{\epsilon }+\frac{(2\beta )^{\frac{p}{2}}}{\hat{\epsilon }^{\frac{p-2}{2}}}\right] ,\,c_2$*and*$c_3$*are positive constants*.

### *Proof*

Applying G-Itôs formula to $|Y(t)|^p,$ for $p\ge 2$, we proceed as follows

6 $$\begin{aligned} E\left[ \sup _{0\le v\le t}|Y(t)|^p\right] &\le {} E|\zeta (0)|^p+pE \left[ \sup _{0\le v\le t}\int _0^t|Y(v)|^{p-1}|\kappa (v,Y_v)|dv\right] \nonumber \\ & \quad + E\left[ \sup _{0\le v\le t}\int _0^tp|Y(v)|^{p-1}|\mu (v,Y_v)|d B(v)\right] \nonumber \\ & \quad+ E\left[ \sup _{0\le v\le t}\int _0^t[p|Y(v)|^{p-1}|\lambda (v,Y_v)|+\frac{p(p-1)}{2}|Y(v)|^{p-2}|\mu (v,Y_v)|^2]d \langle B, B \rangle (v)\right] \nonumber \\ &= E|\zeta (0)|^p+I_1+I_2+I_3, \end{aligned}$$

where

$$\begin{aligned} I_1 &= pE\left[ \sup _{0\le v\le t}\int _0^t|Y(v)|^{p-1}|\kappa (v,Y_v)|dv\right] ,\\ I_2 &= pE\left[ \sup _{0\le v\le t}\int _0^t|Y(v)|^{p-1}|\mu (v,Y_v)|d B(v)\right] ,\\ I_3 &= pE\left[ \sup _{0\le v\le t}\int _0^t\left[ |Y(v)|^{p-1}|\lambda (v,Y_v)|+\frac{(p-1)}{2}|Y(v)|^{p-2}|\mu (v,Y_v)|^2\right] d \langle B, B \rangle (v)\right] . \end{aligned}$$

By non-linear growth condition (3) and Lemma 6, for any $\hat{\epsilon }>0,$ we get

$$\begin{aligned} |Y(t)|^{p-1}|\kappa (t,Y_t)| &\le {} \frac{(p-1)\hat{\epsilon }|Y(t)|^p}{p}+\frac{|\kappa (t,Y_t)|^p}{p\hat{\epsilon }^{p-1}}\\ &\le {} \frac{(p-1)\hat{\epsilon }\Vert Y(t)\Vert ^p}{p}+\frac{\left[ \Upsilon (1+\Vert Y_t\Vert ^2)\right] ^\frac{p}{2}}{p\hat{\epsilon }^{p-1}}\\ &\le {} \frac{(p-1)\hat{\epsilon }\Vert Y(t)\Vert ^p}{p}+\frac{\left[ \alpha +\beta (1+\Vert Y_t\Vert ^2)\right] ^\frac{p}{2}}{p\hat{\epsilon }^{p-1}}. \end{aligned}$$

Using the inequality $(a+b)^p\le 2^{p-1}(a^p+b^p)$ and the fact $\sup _{-\tau \le v\le T}|Y(v)|^p\le \Vert \zeta \Vert ^p+\sup _{0\le v\le T}|Y(v)|^p$, we proceed as follows

$$\begin{aligned} |Y(t)|^{p-1}|\kappa (t,Y_t)| &\le {} \frac{(p-1)\hat{\epsilon }\Vert Y(t)\Vert ^p}{p}+\frac{[\alpha +\beta +\beta \Vert Y_t\Vert ^2]^\frac{p}{2}}{p\hat{\epsilon }^{p-1}}\\ &\le {} \frac{(p-1)\hat{\epsilon }\Vert Y(t)\Vert ^p}{p} +\frac{(2)^{\frac{p}{2}-1}[(\alpha +\beta )^{\frac{p}{2}}+(\beta )^{\frac{p}{2}}\Vert Y_t\Vert ^{p}]}{p\hat{\epsilon }^{p-1}}\\ &\le {} \frac{(p-1)\hat{\epsilon }\Vert Y(t)\Vert ^p}{p} +\frac{(2)^{\frac{p}{2}-1}[(\alpha +\beta )^{\frac{p}{2}}+(\beta )^{\frac{p}{2}}\Vert \zeta \Vert ^p+(\beta )^{\frac{p}{2}}\Vert Y(t)\Vert ^{p}]}{p\hat{\epsilon }^{p-1}}\\ &\le {} \frac{(p-1)\hat{\epsilon }\Vert Y(t)\Vert ^p}{p} +\frac{(2)^{\frac{p}{2}}[(\alpha +\beta )^{\frac{p}{2}}+(\beta )^{\frac{p}{2}}\Vert \zeta \Vert ^p]+(2\beta )^{\frac{p}{2}}\Vert Y(t)\Vert ^{p}}{2p\hat{\epsilon }^{p-1}}\\ &= \frac{(2)^{\frac{p}{2}}[(\alpha +\beta )^{\frac{p}{2}}+(\beta )^{\frac{p}{2}}\Vert \zeta \Vert ^p]}{2p\hat{\epsilon }^{p-1}} +\left[\frac{(p-1)\hat{\epsilon }}{p}+\frac{(2\beta )^{\frac{p}{2}}}{2p\hat{\epsilon }^{p-1}}\right]\Vert Y(t)\Vert ^p, \end{aligned}$$

which yields

7 $$\begin{aligned} pE|Y(t)|^{p-1}|\kappa (t,Y_t)|\le \alpha _1 +\beta _1E\Vert Y(t)\Vert ^p, \end{aligned}$$

where $\alpha _1=\frac{(2)^{\frac{p}{2}}[(\alpha +\beta ))^{\frac{p}{2}}+(\beta )^{\frac{p}{2}}E\Vert \zeta \Vert ^p]}{2\hat{\epsilon }^{p-1}}$ and $\beta _1=(p-1)\hat{\epsilon }+\frac{(2\beta )^{\frac{p}{2}}}{2 \hat{\epsilon }^{p-1}}.$ In a similar fashion as above we get

8 $$\begin{aligned}&p|Y(t)|^{p-1}|\lambda (t,Y_t)|\le \alpha _1 +\beta _1\Vert Y(t)\Vert ^p,\nonumber \\&p|Y(t)|^{p-1}|\mu (t,Y_t)|\le \alpha _1 +\beta _1\Vert Y(t)\Vert ^p. \end{aligned}$$

Next by using Lemma 6, non-linear growth condition (3), inequality $(a+b)^p\le 2^{p-1}(a^p+b^p)$ and the fact $\sup _{-\tau \le v\le T}|Y(v)|^p\le \Vert \zeta \Vert ^p+\sup _{0\le v\le T}|Y(v)|^p$, we have

$$\begin{aligned} |Y(t)|^{p-2}|\mu (t,Y_t)|^2 &\le {} \frac{(p-2)\hat{\epsilon }|Y(t)|^p}{p}+\frac{2|\mu (t,Y_t)|^p}{p\hat{\epsilon }^{\frac{p-2}{2}}}\\ &\le {} \frac{(p-2)\hat{\epsilon }\Vert Y(t)\Vert ^p}{p}+\frac{2[\Upsilon (1+\Vert Y_t\Vert ^2)]^\frac{p}{2}}{p\hat{\epsilon }^{\frac{p-2}{2}}}\\ &\le {} \frac{(p-2)\hat{\epsilon }\Vert Y(t)\Vert ^p}{p}+\frac{2[\alpha +\beta (1+\Vert Y_t\Vert ^2)]^\frac{p}{2}}{p\hat{\epsilon }^{\frac{p-2}{2}}}\\ &\le {} \frac{(p-2)\hat{\epsilon }\Vert Y(t)\Vert ^p}{p}+\frac{2[\alpha +\beta +\beta \Vert Y_t\Vert ^2]^\frac{p}{2}}{p\hat{\epsilon }^{\frac{p-2}{2}}}\\ &\le {} \frac{(p-1)\hat{\epsilon }\Vert Y(t)\Vert ^p}{p} +\frac{(2)^{\frac{p}{2}}[(\alpha +\beta )^{\frac{p}{2}}+(\beta )^{\frac{p}{2}}\Vert Y_t\Vert ^{p}]}{p\hat{\epsilon }^{\frac{p-2}{2}}}\\ &\le {} \frac{(p-2)\hat{\epsilon }\Vert Y(t)\Vert ^p}{p} +\frac{(2)^{\frac{p}{2}}[(\alpha +\beta)^{\frac{p}{2}}+(\beta )^{\frac{p}{2}}\Vert \zeta \Vert ^p+(\beta )^{\frac{p}{2}}\Vert Y(t)\Vert ^{p}]}{p\hat{\epsilon }^{\frac{p-2}{2}}}\\ &\le {} \frac{(p-2)\hat{\epsilon }\Vert Y(t)\Vert ^p}{p} +\frac{(2)^{\frac{p}{2}}[(\alpha +\beta )^{\frac{p}{2}}+(\beta )^{\frac{p}{2}}\Vert \zeta \Vert ^p]+(2\beta )^{\frac{p}{2}}\Vert Y(t)\Vert ^{p}}{p\hat{\epsilon }^{\frac{p-2}{2}}}\\ &= \frac{(2)^{\frac{p}{2}}[(\alpha +\beta )^{\frac{p}{2}}+(\beta )^{\frac{p}{2}}\Vert \zeta \Vert ^p]}{p\hat{\epsilon }^{\frac{p-2}{2}}} +\left[ \frac{(p-1)\hat{\epsilon }}{p}+\frac{(2\beta )^{\frac{p}{2}}}{p\hat{\epsilon }^{\frac{p-2}{2}}}\right] \Vert Y(t)\Vert ^p, \end{aligned}$$

which gives

9 $$\begin{aligned} pE|Y(t)|^{p-2}|\mu (t,Y_t)|^2\le \alpha _2 +\beta _2E\Vert Y(t)\Vert ^p, \end{aligned}$$

where $\alpha _2=\frac{(2)^{\frac{p}{2}}[(\alpha +\beta ))^{\frac{p}{2}}+(\beta )^{\frac{p}{2}}E\Vert \zeta \Vert ^p]}{\hat{\epsilon }^{\frac{p-2}{2}}}$ and $\beta _2=\left[ (p-1)\hat{\epsilon }+\frac{(2\beta )^{\frac{p}{2}}}{\hat{\epsilon }^{\frac{p-2}{2}}}\right] .$ Then $I_1$ can be written as follows

$$\begin{aligned} I_1 &= E\left[ \sup _{0\le v\le t}\int _0^tp|Y(v)|^{p-1}|\kappa (v,Y_v)|dv\right] \\ {} &\le {} \int _0^t [\alpha _1 +\beta _1E\Vert Y(t)\Vert ^p]dv\\ &\le {} \alpha _1T+\beta _1\int _0^t E(\Vert Y(v)\Vert ^p)dv. \end{aligned}$$

By inequalities (8) and the Burkholder–Davis–Gundy (BDG) inequalities (Gao 2009), $I_2$ can be written as follows

$$\begin{aligned} I_2 &= E\left[ \sup _{0\le v\le t}\left|\int _0^t\left[ p|Y(v)|^{p-1}|\lambda (v,Y_v)|+\frac{p(p-1)}{2}|Y(v)|^{p-2}|\mu (v,Y_v)|^2\right] d \langle B, B \rangle (v)\right] \right|\\ &\le {} c_1\int _0^t\left[ pE|Y(v)|^{p-1}|\lambda (v,Y_v)|+\frac{p(p-1)}{2}E|Y(v)|^{p-2}|\mu (v,Y_v)|^2\right] dv\\ &\le {} c_1\int _0^t\left[ \alpha _1 +\beta _1E\Vert Y(t)\Vert ^p+\frac{(p-1)}{2}(\alpha _2 +\beta _2E\Vert Y(t)\Vert ^p)\right] dv\\ &\le {} c_1\left( \alpha _1 +\frac{1}{2}(p-1)\alpha _2\right) T +c_1\left( \beta _1+\frac{1}{2}(p-1)\beta _2\right) \int _0^tE\Vert Y(t)\Vert ^pdv\\ {}&\end{aligned}$$

Next we use the BDG inequalities (Gao 2009), inequality (8), mean value theorem and the inequality $|a||b|\le \frac{a^2}{2}+\frac{b^2}{2}$ as follows

$$\begin{aligned} I_3 &= pE\left[ \sup _{0\le v\le t}\left|\int _0^t|Y(v)|^{p-1}|\mu (v,Y_v)|dB(v)\right|\right] \\ &\le {} pc_3E\left[ \int _0^t|Y(v)|^{2p-2}|\mu (v,Y_v)|^2dv\right] ^{\frac{1}{2}}\\ &\le {} pc_3E\left[ \sup _{0\le v\le t }[|Y(v)|^{p}]^{\frac{1}{2}}\int _0^t|Y(v)|^{p-2}|\mu (v,Y_v)|^2dv\right] ^{\frac{1}{2}}\\ &\le {} \frac{1}{2}E\left[ \sup _{0\le v\le t }|Y(v)|^{p}\right] +\frac{p^2c^2_3}{2}E \left[ \int _0^t|Y(v)|^{p-2}|\mu (v,Y_v)|^2dv\right] \\ &\le {} \frac{1}{2}E\left[ \sup _{0\le v\le t }|Y(v)|^{p}\right] +\frac{pc^2_3}{2}\int _0^t[\alpha _2 +\beta _2E\Vert Y(t)\Vert ^p]dv\\ &\le {} \frac{1}{2} pc^2_3\alpha _2T+\frac{1}{2}E\left[ \sup _{0\le v\le t }|Y(v)|^{p}\right] +\frac{1}{2}pc^2_3\beta _2\int _0^tE\Vert Y(t)\Vert ^pdv \end{aligned}$$

Using the values of $I_1,\,I_2$ and $I_3$ in (2) we get

$$\begin{aligned} E\left[ \sup _{0\le v\le t}|Y(v)|^p\right] &\le \alpha _1T+\beta _1\int _0^t E\left(\Vert Y(v)\Vert ^p\right)dv\\& \quad +\,c_1\left( \alpha _1 +\frac{1}{2}(p-1)\alpha _2\right) T +c_1\left( \beta _1+\frac{1}{2}(p-1)\beta _2\right) \int _0^tE\Vert Y(t)\Vert ^pdv\\& \quad+\,\frac{1}{2} pc^2_3\alpha _2T+\frac{1}{2}E\left[ \sup _{0\le v\le t }|Y(v)|^{p}\right] +\frac{1}{2}pc^2_3\beta _2\int _0^tE\Vert Y(t)\Vert ^pdv\\ &= {} \frac{1}{2}E\left[ \sup _{0\le v\le t }|Y(v)|^{p}+T\left[ \alpha _1(1+c_1) +\frac{1}{2}\alpha _2(c_1(p-1)+pc^2_3)\right] \right. \\& \quad+\,\left[ \beta _1(1+c_1)+\frac{1}{2}\beta _2(p-1+pc^2_3)\right] \int _0^t E(\Vert Y(v)\Vert ^p)dv, \end{aligned}$$

simplification yields,

$$\begin{aligned} E\left[ \sup _{0\le v\le t}|Y(v)|^p\right] &\le {} T\left[ 2\alpha _1(1+c_1) +\alpha _2(c_1(p-1)+pc^2_3)\right] \\& \quad+\,\left[ 2\beta _1(1+c_1)+\beta _2(p-1+pc^2_3)\right] \int _0^t E(\Vert Z(v)\Vert ^p)dv. \end{aligned}$$

By the Gronwall’s inequality

10 $$\begin{aligned} E\left[ \sup _{0\le v\le t}|Y(v)|^p\right] \le \alpha _3e^{\beta _3t}, \end{aligned}$$

where $\alpha _3=T[2\alpha _1(1+c_1) +\alpha _2(c_1(p-1)+pc^2_3)]$ and $\beta _3=[2\beta _1(1+c_1)+\beta _2(p-1+pc^2_3)].$ By taking $t=T,$ we have

11 $$\begin{aligned} E\left[ \sup _{0\le v\le T}|Y(v)|^p\right] \le \alpha _3e^{\beta _3T}. \end{aligned}$$

Noting the fact that $\sup _{-\tau \le v\le T}|Y(v)|^p\le \Vert \zeta \Vert ^p+\sup _{0\le v\le T}|Y(v)|^p$, we proceed as follows

$$\begin{aligned} E\left[ \sup _{-\tau \le v\le T}|Y(v)|^p\right] &\le {} E\Vert \zeta \Vert ^p+E \left[ \sup _{0\le v\le T}|Y(v)|^p\right] \\ &\le {} E\Vert \zeta \Vert ^p+\alpha _3e^{\beta _3T}. \end{aligned}$$

The proof is complete. $\square$

## Continuity of *p*th moment for SFDE in the G-framework

In the next theorem, under non-linear growth condition, it is shown that the *p*th moment of the solution to SFDE in the G-framework (1) is continuous.

### **Theorem 9**

*Assume the non-linear growth condition* (3) *holds. Let*$E\Vert \zeta \Vert ^p<\infty$*and*$p\ge 2$. *Then*

$$\begin{aligned} E[|Y(t)-Y(s)|^p]\le \gamma (t)(t-s)^{p}, \end{aligned}$$

*where*$\gamma (t)=3^{\frac{3p}{2}-2}(1+c_2+c_3) [\alpha ^{\frac{p}{2}}+\beta ^{\frac{p}{2}}+\beta ^{\frac{p}{2}} E\Vert \zeta \Vert ^{p}+ \beta ^{\frac{p}{2}} \alpha _3e^{\beta _3T}],\,c_2,\,c_3,\,\alpha ,\,\beta ,\,\alpha _3$*and*$\beta _3$*are positive constants*.

### *Proof*

By using the inequality $(a+b+c)^p\le 3^{p-1}(a^p+b^p+c^p)$, Eq. (1) follows

$$\begin{aligned} |Y (t)-Y (s)|^p &= {} 3^{p-1}\left| \int _s^t\kappa (q, Y_{q}) dq\right|^p+ 3^{p-1}\left|\int _s^t\lambda (q, Y_{q}) d \langle B, B \rangle (q) \right|^p\\& \quad +\,3^{p-1}\left|\int _s^t\mu (q, Y_{q}) dB(q)\right|^p. \end{aligned}$$

Applying G-expectation on both sides, using the BDG inequalities (Gao 2009), Holder’s inequality and non-linear growth condition, we proceed as follows

$$\begin{aligned} E|Y (t)-Y(s)|^p &\le {} 3^{p-1}(t-s)^{p-1}E \int _s^t|\kappa (q, Y_{q})|^p dq+ 3^{p-1}c_2(t-s)^{p-1}\int _s^t|\lambda (q, Y_{q})|^p dq \\& \quad +\,3^{p-1}c_3(t-s)^{p-1}\int _s^t|\mu (q, Y_{q})|^p dq\\ &\le {} 3^{p-1}(t-s)^{p-1}E \int _s^t\left[ \Upsilon (1+\Vert Y_q\Vert ^2)\right] ^\frac{p}{2} dq+ 3^{p-1}c_2(t-s)^{p-1}\int _s^t\left[ \Upsilon (1+\Vert Y_q\Vert ^2)\right] ^\frac{p}{2} dq \\& \quad +\,3^{p-1}c_3(t-s)^{p-1}\int _s^t[\Upsilon (1+\Vert Y_q\Vert ^2)]^\frac{p}{2} dq\\ &= {} 3^{p-1}(t-s)^{p-1}[1+c_2+c_3]E \int _s^t[\Upsilon (1+\Vert Y_q\Vert ^2)]^\frac{p}{2} dq\\ &\le {} 3^{p-1}(t-s)^{p-1}[1+c_2+c_3]E \int _s^t[\alpha +\beta (1+\Vert Y_q\Vert ^2)]^\frac{p}{2} dq\\ &\le {} 3^{p-1}(t-s)^{p-1}[1+c_2+c_3]E \int _s^t[\alpha +\beta +\beta \Vert Y_q\Vert ^2]^\frac{p}{2} dq\\ &\le {} 3^{p-1}(t-s)^{p-1}[1+c_2+c_3]3^{\frac{p}{2}-1} \int _s^t[(\alpha )^{\frac{p}{2}}+(\beta )^{\frac{p}{2}} +(\beta )^{\frac{p}{2}} E\Vert Y_q\Vert ^{p}] dq\\ &\le {} 3^{p-1}(t-s)^{p-1}[1+c_2+c_3]3^{\frac{p}{2}-1} \int _s^t\left[ \alpha ^{\frac{p}{2}}+\beta ^{\frac{p}{2}} +\beta ^{\frac{p}{2}} E\Vert \zeta \Vert ^{p}+\beta ^{\frac{p}{2}}\sup _{0\le s\le r\le q}E\Vert Y(r)\Vert ^{p}\right] dr\\ &\le {} 3^{\frac{3p}{2}-2}(t-s)^{p}[1+c_2+c_3] \left[ \alpha ^{\frac{p}{2}}+\beta ^{\frac{p}{2}}+\beta ^{\frac{p}{2}} E\Vert \zeta \Vert ^{p}\right] \\&\quad +\,3^{\frac{3p}{2}-2}(t-s)^{p-1}[1+c_2+c_3] \beta ^{\frac{p}{2}} \int _s^t\sup _{0\le s\le r\le q}E\Vert Y(r)\Vert ^{p} dr \end{aligned}$$

By using the inequality (11), it follows

$$\begin{aligned} E|Y(t)-Y(s)|^p &\le {} 3^{\frac{3p}{2}-2}(t-s)^{p}[1+c_2+c_3] \left[ \alpha ^{\frac{p}{2}}+\beta ^{\frac{p}{2}}+\beta ^{\frac{p}{2}} E\Vert \zeta \Vert ^{p}\right] \\& \quad +\,3^{\frac{3p}{2}-2}(t-s)^{p-1}[1+c_2+c_3] \beta ^{\frac{p}{2}} \int _s^t \alpha _3e^{\beta _3T} dr\\ &\le {} 3^{\frac{3p}{2}-2}(t-s)^{p}[1+c_2+c_3] \left[ \alpha ^{\frac{p}{2}}+\beta ^{\frac{p}{2}}+\beta ^{\frac{p}{2}} E\Vert \zeta \Vert ^{p}\right] \\& \quad +\,3^{\frac{3p}{2}-2}(t-s)^{p}[1+c_2+c_3] \beta ^{\frac{p}{2}} \alpha _3e^{\beta _3T} \\ &= {} \gamma (t)(t-s)^{p}, \end{aligned}$$

where $\gamma (t)=3^{\frac{3p}{2}-2}(1+c_2+c_3)[ [\alpha ^{\frac{p}{2}}+\beta ^{\frac{p}{2}}+\beta ^{\frac{p}{2}} E\Vert \zeta \Vert ^{p}+ \beta ^{\frac{p}{2}} \alpha _3e^{\beta _3T}]$. The proof is complete. $\square$

In the above theorem $c_2,\,c_3,\,\alpha ,\,\beta ,\,\alpha _3$ and $\beta _3$ are positive constants. The values of $\alpha _3$ and $\beta _3$ are given in Theorem 8.

## Path-wise asymptotic estimate

Next, by using Theorem 8 we study the path-wise asymptotic estimate for the solution of SFDE in the G-framework (1). It is understood that $\lim _{t\rightarrow \infty }\sup \frac{1}{t}log|Y(t)|$ is the Lyapunov exponent (Kim 2014). It is shown that the *p*th moment of Lyapunov exponent should not be greater than $\frac{1}{p}[2\beta _1(1+c_1)+\beta _2(p-1+pc^2_3)],$ where $c_1,\,c_3,\,\beta _1,\,\beta _2$ are positive constants and $p\ge 2.$

### **Theorem 10**

*Assume that the non-linear growth condition* (3) *holds. Then*

$$\begin{aligned} \lim _{t\rightarrow \infty }\sup \frac{1}{t}log|Y(t)|\le \frac{1}{p}\left[ 2\beta _1(1+c_1)+\beta _2(p-1+pc^2_3)\right] \,\,\,\,\,\,\,q.s. \end{aligned}$$

### *Proof*

For each $k=1,2,\ldots ,$ using the non-linear growth condition in a similar fashion as in Theorem 8, Eq. (10) we obtain,

$$\begin{aligned} E\left( \sup _{k-1\le t\le k}|Y(t)|^p\right) \le \alpha _3 e^{\beta _3 k}, \end{aligned}$$

where $\alpha _3=T[2\alpha _1(1+c_1) +\alpha _2(c_1(p-1)+pc^2_3)]$ and $\beta _3=[2\beta _1(1+c_1)+\beta _2(p-1+pc^2_3)]$. Recall that *E* is a sub-linear expectation. Unlike a classical expectation, it is not based on a particular probability space. So, instead of probability, we use a different concept known as capacity. Thanks to Theorem 7 for any arbitrary $\epsilon >0,$ we have

$$\begin{aligned} \hat{C}\left( w{:}\,\sup _{k-1\le t\le k}|Y(t)|^p>e^{(\beta _3+\epsilon )k}\right) &\le {} \frac{E\left[ \sup _{k-1\le t\le k}|Y(t)|^p\right] }{e^{(\beta _3+\epsilon )k}}\\ &\le {} \frac{\alpha e^{\beta _3 k}}{e^{(\beta _3+\epsilon )k}}\\ &= {} \alpha e^{-\epsilon k}. \end{aligned}$$

The Borel–Cantelli lemma follows for almost all $w\in \Omega$, there exists a random integer $k_0=k_0(w)$ such that

$$\begin{aligned} \sup _{k-1\le t\le k}|Y(t)|^p \le e^{(\beta _3+\epsilon )k} \,\,\,\,\,\,\,\,\,whenever\,\,\,\,k\ge k_0, \end{aligned}$$

consequently, we get

$$\begin{aligned} \lim _{t\rightarrow \infty }\sup \frac{1}{t}log|Y(t)| &\le {} \frac{\beta _3+\epsilon }{p}\\ &= {} \frac{1}{p}\left[ 2\beta _1(1+c_1)+\beta _2(p-1+pc^2_3)\right] +\frac{\epsilon }{p},\,\,\,\, q.s. \end{aligned}$$

But $\epsilon$ is arbitrary, so

$$\begin{aligned} \lim _{t\rightarrow \infty }\sup \frac{1}{t}log|Y(t)| \le \frac{1}{p}\left[ 2\beta _1(1+c_1)+\beta _2(p-1+pc^2_3)\right] ,\,\,\,\, q.s. \end{aligned}$$

The proof is complete. $\square$

### *Remark 11*

In the above theorem if $p=2,$ then

$$\begin{aligned} \lim _{t\rightarrow \infty }\sup \frac{1}{t}log|Y(t)| \le \beta _1(1+c_1)+\frac{1}{2}\beta _2\left( 1+2c^2_3\right) , \end{aligned}$$

Hence $\beta _1(1+c_1)+\frac{1}{2}\beta _2(1+2c^2_3)$ is the upper bound for second moment of Lyapunov exponent.

## Conclusion

Generally, we cannot find explicit solutions to nonlinear SDEs. Thus one needs to present the analysis for solutions to these equations. Existence and moment estimates are the most important characteristics for solutions to SDEs. Here, we have used some important inequalities such as Bihari’s inequality, Hölder’s inequality, Gronwall’s inequality and Burkholder–Davis–Gundy (BDG) inequalities to investigate the *p*th moment estimates for SFDEs driven by G-Brownian motion. Then the asymptotic estimates for these equations have been developed. Furthermore, continuity of *p*th moment for the solutions to SFDEs in the G-framework has been proved. The G-Brownian motion theory is the generalization of the classical Brownian motion theory. The methodology used to estimate *p*th moment for SDE is interesting and applicable in various practical applications. For example, *p*th moment estimates are useful in biological population models (Shang 2013a) and distributed system control (Shang 2012, 2013b, 2015). The methods of the *p*th moment estimation, developed in our paper, can be used to extend the related theory in above mentioned papers.
